# Mechanistic Target of Rapamycin Complex 1 Signaling Links Hypoxia to Increased IGFBP-1 Phosphorylation in Primary Human Decidualized Endometrial Stromal Cells

**DOI:** 10.3390/biom11091382

**Published:** 2021-09-18

**Authors:** Pinki Nandi, Chloe E. Jang, Kyle Biggar, Chidambra D. Halari, Thomas Jansson, Madhulika B. Gupta

**Affiliations:** 1 Departments of Pediatrics, University of Western Ontario, London, ON N6A 5C1, Canada; pnandi@uwo.ca; 2 Department of Biochemistry, University of Western Ontario, London, ON N6A 5C1, Canada; cjang6@uwo.ca; 3 Institute of Biochemistry and Department of Biology, Carleton University, Ottawa, ON K1S 5B6, Canada; kyle_biggar@carleton.ca; 4 Departments of Anatomy and Cell Biology, University of Western Ontario, London, ON N6A 3K7, Canada; chalari@uwo.ca; 5 Department of Obstetrics and Gynecology, Division of Reproductive Sciences, University of Colorado Anschutz Medical Campus, Aurora, CO 80045, USA; thomas.jansson@cuanschutz.edu; 6 Children’s Health Research Institute, London, ON N6C 2V5, Canada

**Keywords:** fetal development, placental insufficiency, humans, insulin-like growth factor I, receptor, IGF Type 1

## Abstract

Insulin-like growth factor-1 (IGF-1) bioavailability in pregnancy is governed by IGF binding protein (IGFBP-1) and its phosphorylation, which enhances the affinity of IGFBP-1 for the growth factor. The decidua is the predominant source of maternal IGFBP-1; however, the mechanisms regulating decidual IGFBP-1 secretion/phosphorylation are poorly understood. Using decidualized primary human endometrial stromal cells (HESCs) from first-trimester placenta, we tested the hypothesis that mTORC1 signaling mechanistically links hypoxia to decidual IGFBP-1 secretion/phosphorylation. Hypoxia inhibited mechanistic target of rapamycin (mTORC1) (p-P70-S6K/Thr389, −47%, *p* = 0.038; p-4E-BP1/Thr70, −55%, *p* = 0.012) and increased IGFBP-1 (total, +35%, *p* = 0.005; phosphorylated, Ser101/+82%, *p* = 0.018; Ser119/+88%, *p* = 0.039; Ser 169/+157%, *p* = 0.019). Targeted parallel reaction monitoring-mass spectrometry (PRM-MS) additionally demonstrated markedly increased dual IGFBP-1 phosphorylation (pSer98+Ser101; pSer169+Ser174) in hypoxia. IGFBP-1 hyperphosphorylation inhibited IGF-1 receptor autophosphorylation/ Tyr1135 (−29%, *p* = 0.002). Furthermore, silencing of tuberous sclerosis complex 2 (TSC2) activated mTORC1 (p-P70-S6K/Thr389, +68%, *p* = 0.038; p-4E-BP1/Thr70, +30%, *p* = 0.002) and reduced total/site-specific IGFBP-1 phosphorylation. Importantly, TSC2 siRNA prevented inhibition of mTORC1 and the increase in secretion/site-specific IGFBP-1 phosphorylation in hypoxia. PRM-MS indicated concomitant changes in protein kinase autophosphorylation (CK2/Tyr182; PKC/Thr497; PKC/Ser657). Overall, mTORC1 signaling mechanistically links hypoxia to IGFBP-1 secretion/phosphorylation in primary HESC, implicating decidual mTORC1 inhibition as a novel mechanism linking uteroplacental hypoxia to fetal growth restriction.

## 1. Introduction

A lack of normal increase in uteroplacental blood flow, which may result in inadequate transfer of oxygen and nutrients to the decidua, is believed to be an early event in the development of fetal growth restriction (FGR). FGR increases the risk of perinatal complications [1] and predisposes the infant for developing diabetes and cardiovascular disease in childhood and adult age [2,3,4]. The mechanisms linking reduced uteroplacental oxygen availability and restricted fetal growth are not well understood. 

Insulin-like growth factor-1 (IGF-1) promotes protein translation, proliferation, and anti-apoptotic activity in most cell types [5,6]. The six IGF binding proteins (IGFBP-1 to -6) differentially regulate IGF-1 bioavailability, prolong half-life of IGF-1, and target the growth factor to specific tissues or inhibit or enhance IGF-1-mediated actions [7,8]. IGFBP-1 inhibits IGF-1-induced DNA synthesis [9], cell proliferation [10], amino acid transport [11], and apoptosis [12], as well as being susceptible to proteolytic cleavage [13]. Although mRNAs for all IGFBPs are detectable in the decidualized endometrium during pregnancy, mRNA expression of IGFBP-1 is higher than for the other IGFBPs [14]. Importantly, IGFBP-1 is a major secretory product of the maternal decidua. As well, the decidua is the primary source of maternal circulating IGFBP-1 [15], which plays a critical role in modulating maternal-fetal resource allocation by regulating IGF-1 bioavailability via its interaction with the IGF-1 receptor (IGF-1R) [16,17]. Locally in the placental barrier, IGFBP-1 inhibits trophoblast invasion both directly [18] and indirectly by sequestering IGF-1, a potent stimulator of trophoblast invasion [19]. 

IGFBPs are highly conserved in the N- and C-terminal regions, which form an IGF-1 binding pocket [20]. Three of the six IGFBPs, IGFBP-1, -3 and -5, are phosphorylated [21]. Most notably, phosphorylation of only IGFBP-1 markedly increases its binding affinity for IGF-1, further limiting IGF-1 bioavailability, inhibiting IGF-1R signaling [22] and thereby suppressing the cellular functions of IGF-1 [9,11,23,24]. Decidua produces IGFBP-1 in both non- and differentially-phosphorylated forms [15]. Using decidua tissue collected from delivery placenta, we have previously reported that human FGR is associated with increased decidual IGFBP-1 phosphorylation, inhibition of mechanistic target of rapamycin complex (mTOR), activated amino acid response (AAR) pathway, and markedly increased expression and activity of decidual protein kinase CK2 (casein kinase) [25]. However, if these signaling events and protein kinase CK2 are mechanistically linked in decidua remains to be established. 

Mechanistic target of rapamycin (mTOR) is a serine/threonine kinase that regulates cell growth and metabolism in response to multiple upstream signals, including growth factors and the availability of oxygen, glucose, and amino acids [26,27,28,29,30]. mTOR functions through two distinct protein complexes, mTOR complex 1 (mTORC1) and complex 2 (mTORC2). When activated, mTORC1 phosphorylates eukaryotic translation initiation factor 4E-binding protein 1 (4E-BP1 at Thr70) and ribosomal protein S6 kinase, 70 kDa, polypeptide 1 (P70-S6K at Thr389), promoting protein translation, lipid biogenesis, and metabolism. 

Previous studies underscore the critical role of mTOR signaling in the developing fetus by using in vitro and in vivo models [31,32,33,34]. mTOR signaling is suggested to be necessary for fetal growth in a mouse model of FGR [35]. Decreased signaling activity of mTOR, and in particular mTORC1, inhibits cell growth and metabolism under stress conditions such as low oxygen tension (hypoxia). Oxygen levels regulate mTORC1 activity through multiple pathways in different tissues [36]. However, these reports do not elucidate how the signaling mechanisms of the maternal unit via decidual IGFBP-1 phosphorylation contribute to the development of FGR.

Tuberous sclerosis TSC1/TSC2 (hamartin/tuberin) complex functions as a heterodimer and has pivotal roles in mediating the effect of growth factors, energy, and oxygen on mTORC1 [37]. TSC2 is the catalytic portion of the complex, while TSC1 is necessary to stabilize TSC2 [38]. TSC2 functions as a GTPase-activating protein (GAP) that catalyzes Ras homolog enriched in brain (Rheb) GTPase activity, which results in mTORC1 signaling inhibition [39]. It is established that hypoxia-induced inhibition of mTOR signaling requires an intact TSC1/2 complex [40]. Thus, silencing of TSC1 is expected to have the same downstream effects as TSC2 silencing. Indeed, TSC1 silencing in hypoxia activates mTORC1 signaling in mouse 3T3 [41] and pulmonary artery smooth muscle cells [42]. Hypoxia can activate TSC1/2 through transcriptional regulation of DNA damage response 1 (REDD1), which increases REDD1 expression, while disruption of REDD1 abrogates the hypoxia-induced inhibition of mTOR [40]. In particular, REDD1 negatively regulates mTORC1 signaling via TSC2, by stimulating the release of TSC2 from its growth factor-induced association with inhibitory 14–3–3 proteins [40], providing an additional rationale for targeting TCS2 rather than TSC1 in the current study.

The mTOR signaling pathway in the fetal liver and decidua serves as a critical hub in the overall homeostatic control of fetal growth [31,43,44]. We were the first to demonstrate that mimicking hypoxia in liver (HepG2) cells triggers pronounced increases in IGFBP-1 phosphorylation at Ser101+Ser98 and Ser169+Ser174 residues that markedly increase IGF-1 affinity (~300-fold) and inhibits IGF-1 stimulated cell growth [32,45]. We also provided novel data to show that mTORC1 inhibition constitutes a mechanistic link between hypoxia, reduced fetal liver IGF-1 bioavailability and FGR [32]. Although inhibition of mTOR signaling in placenta and the fetal liver is believed to contribute to the development of FGR [25,31], whether mTORC1 or other upstream regulators of mTORC1 are involved in regulation of decidual IGF-1 bioavailability in FGR is unclear. Furthermore, IGFBP-1 has consensus phosphorylation sequences not only for CK2, which we reported to be increased in FGR decidua [25], but also protein kinase C (PKC) and protein kinase A (PKA) [46]. Autophosphorylation of CK2 at Tyr182 [47] and of PKC at Thr497 [48] as well as Ser657 [49] has been reported to account for functional activation of these kinases. It remains unknown if decidual protein kinase CK2 and PKC are activated in conjunction with inhibition of mTORC1 in response to hypoxia.

During early pregnancy, human endometrial stromal cells (HESC) undergo morphological and functional transformation into decidual parenchymal cells in response to estrogen and progesterone, which remain highly elevated throughout pregnancy [50]. Successful decidualization of human HESC has previously been achieved using 8-bromoadenosine-3′,5′-cyclic monophosphate (8-Br-cAMP) and medroxyprogesterone-acetate (MPA) [51]. 

Applying similar approaches in human immortalized endometrial stromal cells (HIESCs) in vitro [52], we have shown an association of mTOR inhibition with IGFBP-1 hyperphosphorylation using rapamycin [43]. While rapamycin has been regarded as a relatively specific inhibitor of mTORC1 [53], it has become clear that the drug also may inhibit mTORC2 in some cells, in particular following longer exposures and at higher concentrations [54]. Additionally, rapamycin only partially inhibits mTORC1 [55]. Moreover, immortalized cell lines may lack genetic fidelity compared to primary HESC. Thus, studies in cell lines may provide information that is not entirely physiologically relevant and primary cells are likely to better represent the biology of cells in vivo. Hence, molecular mechanisms governing secretion and phosphorylation of IGFBP-1 in human decidua are not yet established and the role of decidual IGFBP-1 hyperphosphorylation in FGR development remains unknown.

Prior studies in the literature using HESC have investigated IGFBP-1 regulation during the decidualization process [56,57]. In contrast, in the current report we focus uniquely on signaling mechanisms in fully decidualized endometrial stromal cells. Our rationale for this is that decidualized HESC have differentiated profiles of signaling regulation, transcription factors, and hormones/growth factors [58,59,60], enabling their indispensable role to establish and maintain pregnancy. In addition, fully decidualized endometrium have unique signaling mechanisms in response to stress stimuli [61]. This is relevant to understanding the role and regulation of decidual IGFBP-1 phosphorylation in FGR since the level of decidual-secreted phosphorylated IGFBP-1 in maternal circulation and amniotic fluid [13,15] increases significantly after complete differentiation of the decidua [15,62].

Therefore, the novelty of this study is that we uniquely apply siRNA gene silencing of TSC2 in combination with hypoxia in primary HESC isolated from first-trimester placenta from human pregnancy. TSC2 is a subunit of the TSC1/2 complex that negatively regulates mTORC1 signaling [37]. Using TSC2 siRNA with primary decidualized HESC, we test the hypothesis that mTORC1 signaling mechanistically links hypoxia to decidual IGFBP-1 secretion and phosphorylation. Furthermore, we use targeted parallel reaction monitoring (PRM-MS) as an innovative approach to identify relative changes in phosphorylation at novel and/or dually phosphorylated IGFBP-1 sites in primary decidualized cells. PRM-MS is used to determine the functional status of protein kinases via their activation (autophosphorylation) in primary decidualized HESC in response to hypoxia and TSC2 silencing.

## 2. Materials and Methods

### 2.1. Ethical Approval

The University of Western Ontario Review Board (REB # 114343) approved the experimental protocols. Placentas were collected with informed consent at termination of uncomplicated 6–9-week pregnancies for non-medical reasons. The maternal age range was between 22–33 years of age. No clinical characteristics of the study subjects were collected except the age and gestational age at termination.

### 2.2. Isolation and Culture of Primary HESC from Termination Placenta

Primary HESC were isolated from the decidual tissues of 6–9-week gestation placenta in accordance to methods described by Halari et al., 2020 [63]. In brief, pooled decidual tissues from 3 placentas (weighing in total approximately 10 g) were inspected, and then visible chorionic villi or fetal membranes were removed. The tissues were rinsed with Hank’s balanced salt solution (HBSS; Gibco; Waltham, MA, USA), followed by Ca^2+^ and Mg^2+^ free HBSS (Gibco) for each isolation. The tissues were incubated at room temperature for 2 h in HBSS, 2 mM sodium bicarbonate; (Sigma Aldrich, St Louis, MO, USA), Gentamicin and Amphotericin B (Gibco), minced and subsequently incubated for 15 min at 37 °C in HBSS containing 0.02% trypsin (Gibco). The tissues were digested with collagenase/hyaluronidase (StemCell Technologies, Vancouver, BC, Canada), 1 mg/mL DNase I (Sigma Aldrich) and penicillin/streptomycin (Gibco) in 1:1 DMEM/F12 medium (Gibco), filtered using a mesh strainer. The recovered cell suspension was centrifuged (1900 rpm, 5 min), washed and subjected to ammonium chloride (StemCell Technologies) for 15 min on ice to lyse red blood cells. The cell suspension was filtered through a 100 μm nylon mesh (Miltenyi Biotec, Bergisch Gladbach, Germany) to obtain a single-cell suspension. This cell suspension was slowly layered on top of histopaque (Sigma Aldrich) and centrifuged in a swinging bucket rotor at 700× *g* for 20 min at room temperature. Afterwards, decidual cells were collected, from the opaque interface, washed, and centrifuged. Isolated primary HESCs were resuspended, seeded and grown in phenol red-free DMEM/F12 medium enriched with 10% heat-inactivated fetal bovine serum (FBS; Gibco), and 1% penicillin/streptomycin (pen/strep) (Gibco). Cells were passaged via trypsinization prior to reaching confluency and were kept in a humidified incubator maintained at 37 °C and 5% CO_2_ levels.

### 2.3. In Vitro Decidualization of HESCs

**Figure 1:**
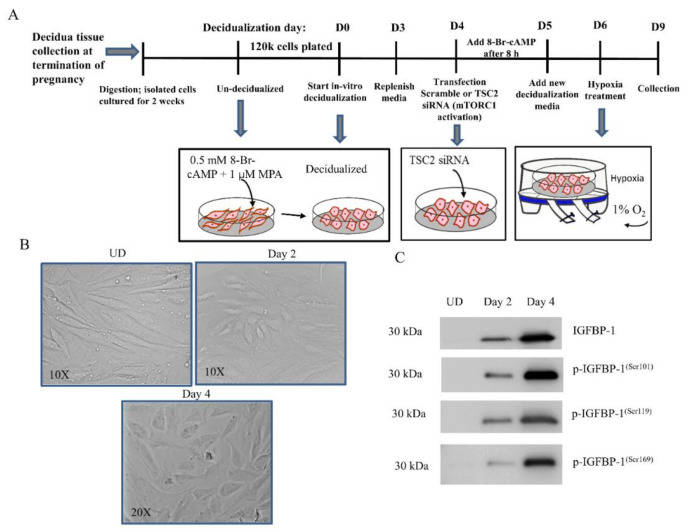
Evaluation of decidualization of HESCs isolated from first-trimester placenta. Primary HESC decidualization and experimental strategy (**A**). Morphological change of primary HESCs after 4 days of decidualization via supplementation with combined 8-Br-cAMP + MPA (**B**). Un-decidualized (UD) primary HESCs maintained an elongated spindle shape throughout the culture period (B-UD, 10×). Cells treated with 8-Br-cAMP + MPA acquired an ovoid-shaped morphology indicative of decidualization at (B-Day 2, 10×) and (B-Day 4, 20×). Total IGFBP-1 secretion and phosphorylation at Ser101, Ser119, and Ser169 were detectable at 48 h treatment of cells with 8-Br-cAMP + MPA and reached a maximum peak by 4 days (**C**). Western blot analysis using equal aliquots of cell media (36 µL) of HESCs (UD) control (*n* = 3) and 8-Br-cAMP + MPA-treated cells (C, Day 2, Day 4) (*n* = 3) for 4 days showed decidualization of primary HESCs concomitantly increased IGFBP-1 phosphorylation at all three serine sites.

A schematic diagram of the culture procedure and protocol for decidualization of HESCs is depicted in Figure 1A. Isolated primary HESCs (between passages two and five) were cultured in control or decidualization media until confluent. The control medium consisted of basal DMEM/F12 supplemented with 2% charcoal-stripped FBS and 1% pen/strep. The decidualization medium included control medium plus 1 µM MPA (Pfizer Canada, Kirkland, QC, Canada) and 0.5 mM 8-br-cAMP (a stable, cell permeable analog of cAMP; Cayman Chemicals, Ann Arbor, MI, USA). Conditioned media (CM) was replenished every 48 h. Cells were allowed to grow until the end of experimental treatment. CM from decidualized and un-decidualized HESCs were collected and, using Western blot, IGFBP-1 levels were detected using rabbit polyclonal IGFBP-1 antibody (a kind gift from Dr. Robert Baxter, Sydney, Australia). Phospho IGFBP-1 was assessed using custom made IGFBP-1 phospho site-specific (Ser101, 119 and 169) antibodies, validated as described previously [22,31].

### 2.4. Assessment of Morphological Changes in Primary Decidualized HESCs

Primary HESCs were assessed daily for morphological differentiation and changes were captured with a phase contrast microscope (Leica Microsystems, Wetzlar, Germany). Fully decidualized cells were used for subsequent treatments.

### 2.5. Hypoxia Treatment of Primary Decidualized HESCs

Fully decidualized HESCs (as described above) were subjected to overnight starvation (0% FBS) before being treated with either hypoxia (1% O_2_) or normoxia (incubator air) for 72 h (Figure 1A), as we optimized previously [45]. Four independent experiments were performed from primary HESCs isolated from 4 sets of three placentas pooled from different study subjects.

For the hypoxia treatment, cells were placed in a hypoxic chamber (Billups-Rothenburg Inc. San Diego, CA, USA) which was flushed with a 1% O_2_, 5% CO_2_, balanced N_2_ gas mixture (Linde Canada Inc., Mississauga, ON, Canada) for 15 min to ensure saturation [45]. The cells in the sealed chamber were placed in a tissue culture incubator at 37 °C. Both CM and cell lysate were collected after 72 h of hypoxic treatment and stored at −80 °C.

### 2.6. RNA Interference-Mediated Silencing in Decidualized HESCs

Gene silencing in decidualized primary HESCs was achieved using transfection with 100 nM small interfering RNA (siRNA) (Sigma-Aldrich) and Dharmafect transfection reagent 4 (Horizon Discovery, Waterbeach, UK) as described in detail previously [32]. As shown in schematics (Figure 1A), 8-Br-cAMP was added back to the transfection media after 8 h and cells left in transfection media for another 16 h. Next, transfection media was replaced with decidualization media and cultured for 24 h in normoxia (20% O_2_), followed by an additional 72 h either in normoxia or in hypoxia (1% O_2_). The TSC2 silencing efficiency was assessed using expression of TSC2 protein and at functional level by examining changes in phosphorylation of mTORC1 downstream targets, P70-S6K at Thr389 and 4E-BP1 at Thr70, using Western blot.

### 2.7. SDS PAGE and Western Blot Analysis

In brief, for Western blot, CM was collected and cell lysates were centrifuged at 13,000 rpm for 30 min, supernatants were collected and stored at −80 °C until used. IGFBP-1 being a secretory protein, the total and phosphorylated IGFBP-1 were measured by loading equal aliquot of CM obtained from an equal number of cells plated to ensure equal loading on SDS PAGE as described previously [31,32,54,64] and then transferred onto a nitrocellulose membrane (Bio-Rad Laboratories, Mississauga, ON, Canada). Membranes were blocked with 5% skim milk for 1 h at room temperature. Total IGFBP-1 and phospho Ser 101, 119, and 169 were assessed as described earlier using phospho-site specific antibodies [22,31].

To determine the expression and phosphorylation of P70–S6K at Thr389, 4E-BP1 at Thr70 and TSC2 [64,65], we used equal cell lysate proteins (30 µg). The primary antibodies were from Cell Signaling Technologies (Danvars, MA, USA) used at a dilution of 1:1000 unless specified otherwise. Secondary antibodies were peroxidase-labeled goat-anti mouse or goat-anti-rabbit antibodies at 1:10,000 dilution (Bio-Rad Laboratories, Mississauga, ON, Canada). Bands were visualized using Clarity Western ECL substrate, imaged using VersaDoc Imaging System and Quantity One imaging software (Bio-Rad Laboratories), and blots were subsequently analyzed using Image Lab software (Bio-Rad Laboratories). The mean density of the control sample bands was assigned an arbitrary value of 1. All individual densitometry values were expressed relative to this mean. IGF-1R activation assay.

### 2.8. PRM-MS Analyses of IGFBP-1 Phosphopeptides and Kinase Autophosphorylation in Decidualized Primary HESCs

#### 2.8.1. Sample Preparation-Immunoprecipitation of IGFBP-1 for Parallel Reaction Monitoring Mass Spectrometry (PRM-MS) Analysis

Four independent experiments were performed, where each experiment utilized freshly extracted HESCs from a pooled sample of *n* = 3 placentas. Equal aliquots of samples from in vitro decidualized cells were used. Decidualized primary HESCs either incubated under hypoxic conditions or silenced for TSC2 (siRNA) with normoxia or hypoxia were pooled and enriched (5-fold) using Amicon Ultracell 10 K MWCO centrifugal filter devices (Sigma Aldrich) and buffer-exchanged with PBS (Gibco). Samples were immunoprecipitated (IP) with well-established IGFBP-1 mouse monoclonal anti-human (mAb) 6303 IGFBP-1 antibody (Medix Biochemica) [43] using Protein A Sepharose beads (50 µL, 50% slurry; GE Healthcare Bio Sciences AB, Uppsala, Sweden), as described previously [32]. The IP samples were washed and then a small aliquot was analyzed by Western blot using anti-human IGFBP-1 polyclonal antibody or anti-human IGFBP-1 phospho Ser101, Ser119, and Ser169 antibodies. This preliminary step confirmed the validity of IP procedure to determine that IGFBP-1 was IP in the samples further used for parallel reaction monitoring mass spectrometry (PRM-MS). The remainder of the IP samples were digested in-solution as described below for PRM-MS analysis. IGFBP-1 also co-immunoprecipitated protein kinases CK2 and PKC which were monitored to determine their activation status.

#### 2.8.2. PRM-MS Analyses of IGFBP-1 Phosphopeptides and Protein Kinase Autophosphorylation

In-solution digestion of the IP samples for PRM-MS analyses of IGFBP-1 phosphopeptides were performed, as described previously [66], to analyze the site-specific phosphorylation of IGFBP-1. IGFBP-1 co-immunoprecipitated CK2 and PKC. The co-IP samples were assessed for kinase autophosphorylation status. Protein samples, pooled from three different biological replicates, were digested first with Asp-N (Roche Diagnostics, Laval, QC, Canada) overnight at 37 °C, then subsequently digested with trypsin (Roche Diagnostics) overnight at 37 °C. Peptide digests were desalted with C18-ZipTip, dried in a Thermo SpeedVac, and then samples were loaded onto a Thermo Easy-Spray analytical column with an Easy-nLC 1000 chromatography pump. A Q-Exactive hybrid quadrupole-Orbitrap mass spectrometer coupled to an Easy-nLC 1000 system (ThermoFisher) was used to collect mass spectra as described [66]. Internal peptide for IGFBP-1 (NH2-ALPGEQQPLHALTR-COOH), CK2 (NH2-WERFVHSENQHLVSPEAL-COOH), were used to normalize respective phosphopeptide data against total protein abundance. The isolation list (not shown) with the Mass [m/z] and the sequences of the peptides used to identify CK2, PKC and IGFBP-1 by PRM-MS were recorded. With two possible IGFBP-1 phosphorylation sites (dual), specific transitions were used to distinguish single-site-phosphorylation from each other (specifically, y14, b6, and b9 ions for pSer169 and pSer174; y12 and b15 ions for pSer98 and pS101). PRM-MS results were analyzed using Skyline software [67]. Data are presented as total peak area relative to the appropriate control group.

### 2.9. IGF-1R Activation Assay

#### 2.9.1. Sample Preparation-Immunoenzymometric Assay (IEMA)

Samples of CM from decidualized primary HESCs grown either under normoxia or hypoxia were collected to perform quantitation of total IGFBP-1 using IGFBP-1 using Immunoenzymometric Assay (IEMA) (Medix Biochemica, Kauniainen, Finland), against IGFBP-1 standard concentrations provided by the assay kit. Prior to IEMA quantitation, CM samples were first buffer exchanged with specialized cell media (high glucose DMEM with sodium pyruvate; Gibco). The buffer exchanged CM was enriched (10-fold) using Amicon Ultracell 10 K MWCO centrifugal filter devices (Sigma Aldrich) and used to perform IEMA as per manufacturer’s instructions.

#### 2.9.2. IGF-1 Induced IGF-1R Autophosphorylation

The IGF-1 bioactivity in CM samples was performed using P6 cells (human IGF-1R overexpressing mouse fibroblast, P6 cells (a BALB/c3T3 cell derivative) (a kind gift from Dr. Renato Baserga, Thomas Jefferson University). The functional assay to determine IGF-1 bioactivity was performed as described previously by assessing IGF-1R autophosphorylation. In brief, samples of CM from decidualized HESCs treated with normoxia or hypoxia with equal amounts of total IGFBP-1 (50 ng) (IEMA) were incubated with 1 ng of recombinant human IGF-1 for 2 h at room temperature on a rotating shaker. The IGFBP-1-IGF-1 complex formed was then used to treat serum starved P6 cells in culture to activate IGF-1R for 10 min at room temperature. CM with IGF-1 alone served as positive control (Control 1, 100% receptor activation), whereas media alone (without IGF-1) served as a negative control. Media with IGFBP-1 (50 ng) from primary decidualized (HESCs) cells under normoxia were considered as Control 2, which was compared to IGFBP-1 (50 ng) from hypoxia treated primary decidualized HESCs sample. The treatment was terminated by aspirating the P6 CM and, subsequently, attached P6 cells were lysed using cell lysis buffer (Cell Signaling Technologies) containing protease and phosphatase inhibitors (Sigma) as described earlier [31]. Changes in IGF-1R autophosphorylation were measured on immunoblot using anti phospho-IGF-1Rβ at Tyr1135 antibody (Cell Signaling Technologies). Subsequently, membrane was stripped and re-probed with total anti IGF-1Rβ (Santa Cruz Biotechnology, Dallas, Texas, USA). The phosphorylated IGF-1Rβ band was normalized to the band intensity of total IGF-1Rβ and finally to β-actin.

### 2.10. Data Presentation and Statistics

Four independent experiments were performed, as described earlier, where each experiment utilized freshly extracted HESCs from a pooled sample of *n* = 3 placentas. Data were analyzed using GraphPad Prism 6 (GraphPad Software, San Diego, CA, USA). The mean density of the control sample bands was assigned an arbitrary value of 1 and treatments, and all individual densitometry were expressed relative to this mean. Every quantified experiment was performed at minimum in triplicate. Statistical significance was determined using student’s paired t-test or one-way ANOVA using Tukey’s multiple comparisons test for post-hoc analysis, as appropriate. Data are presented as the mean + SEM and significance was accepted at *p* < 0.05.

## 3. Results

### 3.1. Changes in Morphology Were Evident Following Decidualization of HESCs Isolated from First-Trimester Placenta

Primary HESCs extracted from first-trimester placenta were purity-checked with vimentin staining (data not shown) and treated with 8-Br-cAMP and MPA for four days to induce decidualization in vitro (Figure 1A). Images were recorded every other day using bright-field microscopy from *n* = 20 fields of view. Successful decidualization was evidenced by the appearance of decidual morphology (Figure 1B). Un-decidualized (UD) confluent primary HESCs maintained their spindle-shaped fibroblast-like morphology throughout the culture period. Treatment with 8-Br-cAMP + MPA induced morphological changes from Day 2, and decidualized cells were recognized by their ovoid-morphology organized in a pavement-like arrangement (Figure 1B, Days 2 and 4).

### 3.2. Decidualization of HESCs Isolated from First-Trimester Placenta Increased IGFBP-1 Secretion/Phosphorylation

As shown by Western blot analysis, decidualization resulted in markedly increased secretion of IGFBP-1 and phosphorylation at key functional serine residues (Ser101, Ser119, Ser169) (Figure 1C) which reached peak levels by four days of culture and plateaued in subsequent days (not shown). These data provide evidence of successful culture and complete decidualization of HESCs by Day 4 and considered appropriate to be used for subsequent experiments.

### 3.3. Hypoxia Inhibited mTORC1 Signaling and Increased Phosphorylation of IGFBP-1 in Decidualized HESCs

**Figure 2:**
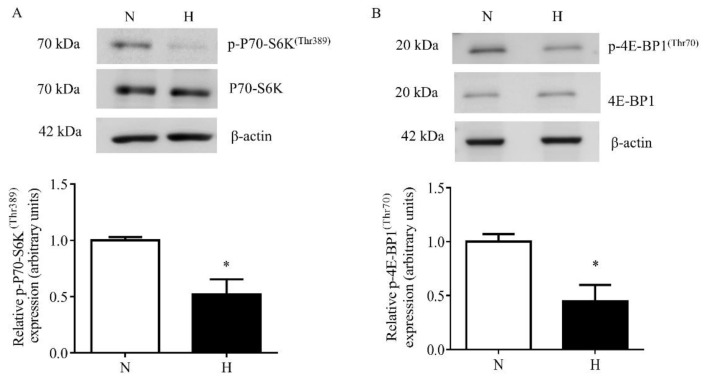
The effect of hypoxia on mTORC1 signaling in decidualized primary HESCs. Representative Western blot of p-P70–S6K^(Thr389)^ (**A**) and p-4E-BP1^(Thr70)^ (**B**) using cell lysates from decidualized primary HESCs cultured under control normoxia (N) (incubator air) or hypoxic (H) (1% O_2_) conditions. Equal amounts of protein from cell lysates were used (30 µg). Levels of phosphorylation were normalized to total protein and β-actin as the loading control. The Western blot data are summarized in the bar graphs (*n* = 4). The data is represented as the mean + SEM; * *p* < 0.05 versus control is considered as per Student’s paired *t*-test.

Primary HESCs after four days of in vitro decidualization were treated with hypoxia for 72 h. As shown in Figure 2, hypoxia alone markedly inhibited the activity of mTORC1 as indicated by a decrease in P70-S6K phosphorylation (Thr389) (–47%, *p* = 0.038) and 4E-BP1 phosphorylation (Thr70) (–55%, *p* = 0.012) (Figure 2A,B).

**Figure 3:**
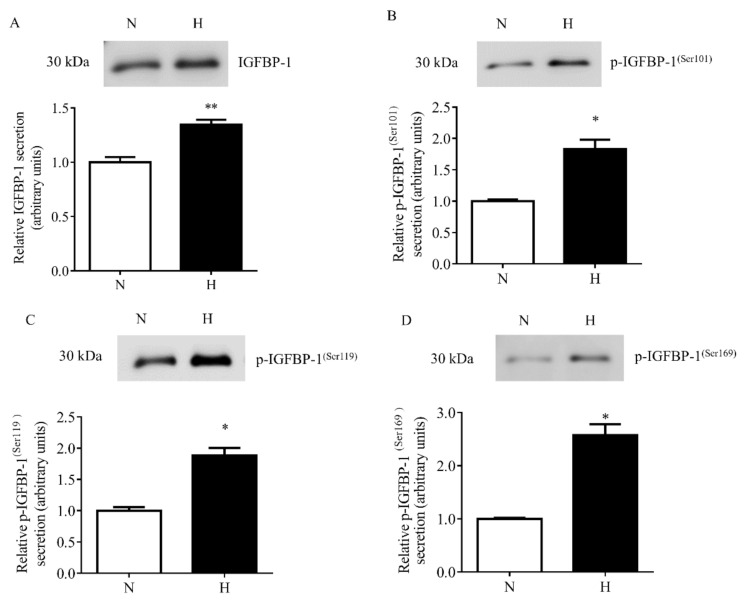
The effect of hypoxia on IGFBP-1 secretion and phosphorylation in decidualized primary HESCs. Representative Western blot of total and phosphorylated IGFBP-1 secreted by decidualized primary HESCs under control normoxia (N) (incubator air) or hypoxic (H) (1% O_2_) conditions (*n* = 4 each). Western blot analyses were performed for total (**A**) and pIGFBP-1^(Ser101)^ (**B**), pIGFBP-1^(Ser119)^ (**C**), and pIGFBP-1^(Ser169)^ (**D**), using equal volume of conditioned media (CM) (36 µL) from control and hypoxia treated cells. The Western blot data are summarized in the bar graphs. Values are given as mean + SEM. *, *p* < 0.05; **, *p* = 0.001–0.05 versus control as per Student’s paired *t*-test.

Further, the hypoxia-treated decidualized primary HESCs were also tested for IGFBP-1 secretion and phosphorylation in CM by Western blot analysis. Hypoxia induced total IGFBP-1 secretion (+35%, *p* = 0.005) as well as IGFBP-1 phosphorylation at three phosphorylation sites, Ser101 (+82%, *p* = 0.018), Ser119 (+88%, *p* = 0.039), and Ser169 (+157%, *p* = 0.019) compared to decidualized HESCs cultured under normoxia (Figure 3A–D).

### 3.4. IGFBP-1 Is Dually Hyperphosphorylated at pSer169+pSer174 in Decidualized HESCs in Response to Hypoxia

**Figure 4:**
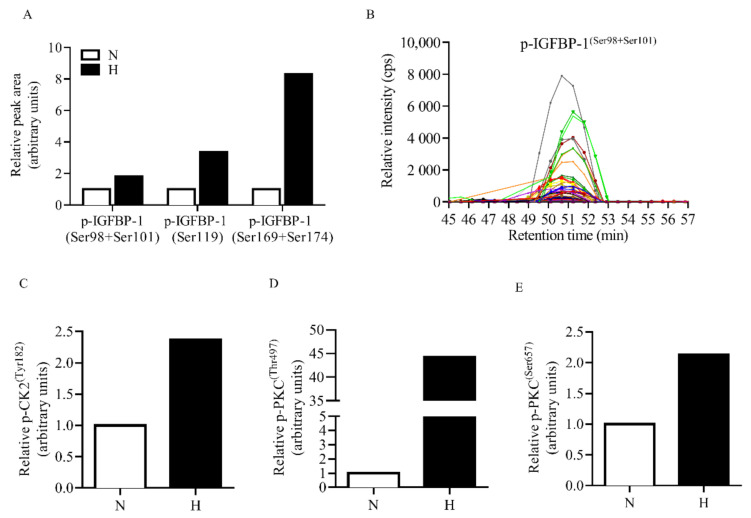
PRM-MS to monitor relative site-specific phosphorylation in hypoxia. Relative phosphorylation of IGFBP-1 (**A**) from normoxic (N) (incubator air) and hypoxic (H) (1% O_2_) conditions in immunoprecipitated (IP) samples using IGFBP-1 antibody. (**B**) PRM-MS transitions are shown in a representative chromatograph used to monitor phosphorylation of IGFBP-1. Peak retention time of IGFBP-1-specific peptides shown in color indicate that transition ions result from the same parent peptide. (Please refer to Supplemental Table for transition list). Phosphorylation of IGFBP-1 co-immunoprecipitated CK2 and PKC kinases was also monitored to determine activation status; p-CK2^(Tyr182)^ (**C**), p-PKC^(Thr497)^ (**D**), and p-PKC^(Ser657)^ (**E**) were individually monitored in control and hypoxic conditions. Data was collected using a Q-Exactive hybrid quadrupole-Orbitrap mass spectrometer. Values are displayed as relative peak area of total detected transitions and normalized by an internal IGFBP-1 peptide.

PRM-MS analysis was performed using decidualized HESCs treated with hypoxia for detection of novel or dually phosphorylated IGFBP-1 sites. These sites could not be detected by Western blot analysis due to the lack of availability of specific antibodies. Hypoxic treatment resulted in markedly increased IGFBP-1 phosphorylation at dual site pSer169+Ser174 (+731%) as compared to normoxia (Figure 4A). The relative changes in pSer119 (+236%) singly and dually at pSer98+Ser101 (+81%) were not as pronounced in IP samples using IGFBP-1 antibody. Peak retention time with a representative chromatograph (pSer98+Ser101) (Figure 4B) confirms that transition ions result from the same parent peptide. Overall while there is increase in phosphorylation at all three serine residues, PRM-MS data show for the first time that hyperphosphorylation at pSer174, in combination with pSer169, may be uniquely involved in regulation of IGFBP-1 phosphorylation.

### 3.5. Increased Protein Kinase Autophosphorylation (Activation) in Decidualized HESCs in Response to Hypoxia

Targeted PRM-MS analysis of autophosphorylation of CK2 and PKC kinases was monitored in decidualized HESC samples co-immunoprecipitated with IGFBP-1. We determined the activation status of CK2 and PKC individually in normoxic control and hypoxic conditions. Hypoxia caused a markedly increased autophosphorylation of CK2 at Tyr182 (+137%), PKC at Thr497 (+4426%), and PKC at Ser657 (+213%), indicating these kinases are activated under hypoxia (Figure 4C–E).

### 3.6. IGFBP-1 Hyperphosphorylation Induced by Hypoxia in Decidualized HESCs Resulted in Inhibition of IGF-1 Bioactivity

**Figure 5:**
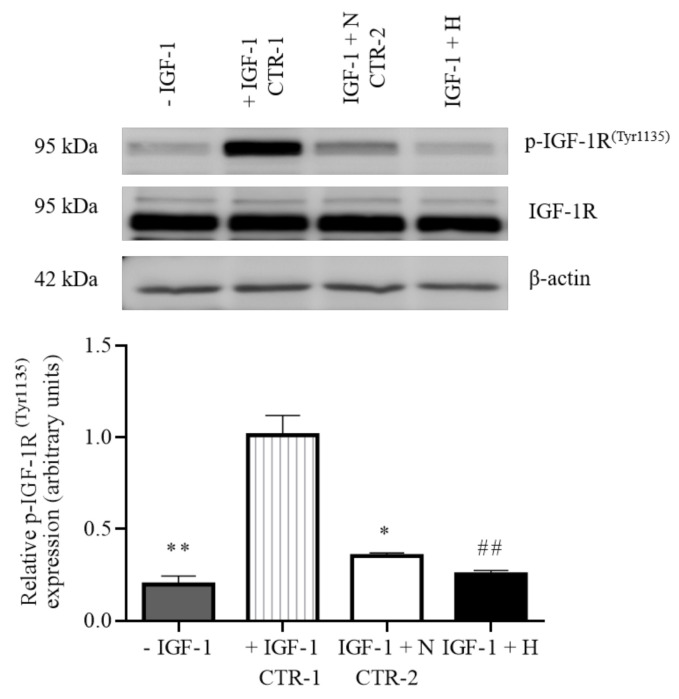
Hypoxia treatment inhibits IGF-1R activity in decidualized primary HESCs. CM was collected from decidualized primary HESCs treated under normoxic (N) (incubator air) or hypoxic (H) (1% O_2_) conditions for 72 h and buffer exchanged with high glucose DMEM media. Equal total IGFBP-1 (50 ng) in the buffer exchanged media were incubated with recombinant human IGF-1 (1 ng) and used to treat P6 cells in culture. P6 cells were lysed and Western blotting was performed (equal loading, 30 µg protein) to determine changes in IGF-1-induced IGF-1R autophosphorylation ^(Tyr1135)^ compared to IGF-1 Control 1 (CTR-1). Media with IGFBP-1 (50 ng) from primary decidualized (HESCs) cells under normoxia was considered as Control 2 (CTR-2). The Western blot data are summarized in the bar graphs (*n* = 4). Data was first normalized to total IGF-1R followed by β-actin. Values are given as mean + SEM. *, *p* < 0.05; ** or ^##^, *p* = 0.001–0.05 versus control as per one-way ANOVA. Tukey’s multiple comparisons test. * = significant difference compared to IGF-1 positive control (CTR-1); # = significant difference compared to conditioned sample control (CTR-2).

The design of the IGF-1R autophosphorylation assay allowed us to distinguish the functional effects of specifically IGFBP-1 hyperphosphorylation on IGF-1 bioactivity in response to hypoxia. CM with only IGF-1 but without IGFBP-1 positive control (Control 1) significantly stimulated IGF-1R autophosphorylation in P6 cells, (+410%, *p* = 0.005) compared to no IGF-1 (negative control) (Figure 5). CM from HESCs under normoxia showed a reduction in IGF-1R autophosphorylation at Tyr1135 compared to Control 1 (−65%, *p* = 0.024). This is because total (non/less phosphorylated) IGFBP-1 also binds IGF-1 but to a lesser extent. When CM from hypoxia-treated cells was added to cultured P6 cells, IGF-1-induced IGF-1R autophosphorylation was significantly reduced in comparison to P6 cells treated with CM samples from HESCs cultured under normoxic conditions (Control 2) (−29%, *p* = 0.002). These data are consistent with the interpretation that the IGFBP-1 hyperphosphorylation in response to hypoxia more prominently inhibits IGF-1 function.

### 3.7. TSC2 siRNA Efficiently Reduced the Expression of the Target Protein TSC2

**Figure 6:**
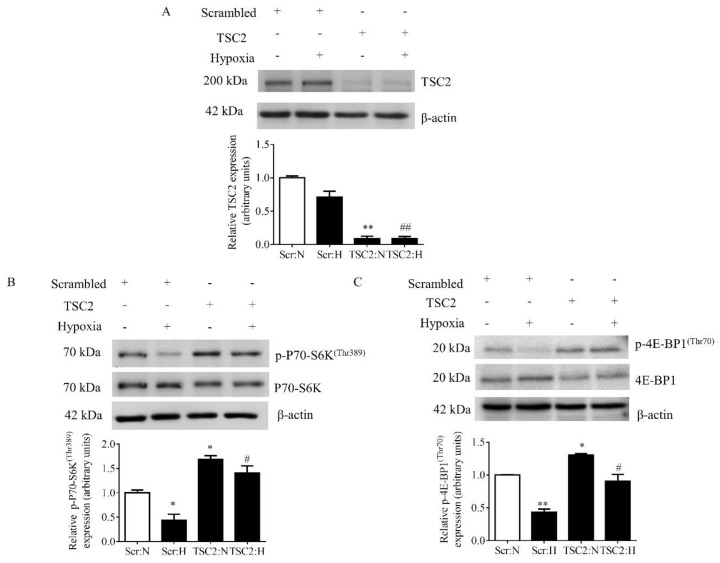
The effect of TSC2 silencing in hypoxia on mTORC1 functional readouts in decidualized primary HESCs. Decidualized primary HESCs were transfected with TSC2 or Scramble (Scr) siRNA, and then cells were cultured in normoxia (N) (incubator air) or in hypoxia (H) (1% O_2_). Representative Western blots validate successful knock down of TSC2 (**A**) and depict the levels of phosphorylation of mTORC1 functional readouts: p-P70-S6K^(Thr389)^ (**B**) and p-4E-BP1^(Thr70)^ (**C**). Equal amounts of protein from cell lysates were used (30 µg). The Western blot data are summarized in the bar graphs (*n* = 4). Levels of phosphorylation were normalized to total protein then β-actin. Values are displayed as mean + SEM; * or ^#^, *p* < 0.05; ** or ^##^, *p* = 0.001–0.05 versus control as per one-way ANOVA. Tukey’s multiple comparisons test. * = significant difference compared to HESCs transfected with Scr siRNA in normoxia; # = significant difference compared to HESCs transfected with Scr siRNA in hypoxia.

To demonstrate that mTORC1 signaling is required in mediating the effects of hypoxia on IGFBP-1, we silenced the endogenous mTORC1 inhibitor TSC2 to specifically activate mTORC1. To confirm that the siRNA reduced expression of the target protein TSC2, encoded by the TSC2 gene, we performed Western blots using cell extracts from siRNA-transfected decidualized HESCs. Markedly decreased protein expression of TSC2 (−91%, *p* = 0.001) confirmed high silencing efficiency of TSC2 (Figure 6A).

### 3.8. TSC2 Silencing in Decidualized HESCs Increased mTORC1 Signaling Activity in Normoxia

Efficient silencing of TSC2 led to significant activation of mTORC1 signaling in decidualized HESCs. Increase in mTORC1 signaling activity was evidenced by significant increase in phosphorylated P70-S6K at Thr389 (+68%, *p* = 0.038) and phosphorylated 4E-BP1 at Thr70 (+30%, *p* = 0.002) with TSC2 siRNA, compared to Scr siRNA in normoxia (Figure 6B,C; lanes 1 and 3).

### 3.9. TSC2 Silencing in Decidualized HESCs in Hypoxia Prevented the Inhibition of mTORC1 Signaling Activity in Response to Hypoxia

TSC2 silencing in hypoxia maintained mTORC1 signaling activity almost to the level observed under normoxia in decidualized HESCs (Figure 6B,C; lanes 3 and 4). These data demonstrate that TSC2 silencing prevented mTORC1 inhibition caused by hypoxia in decidualized primary HESCs.

### 3.10. TSC2 Silencing Prevented the Induction of Total and Phosphorylated IGFBP-1 in Decidualized HESCs in Hypoxia

**Figure 7:**
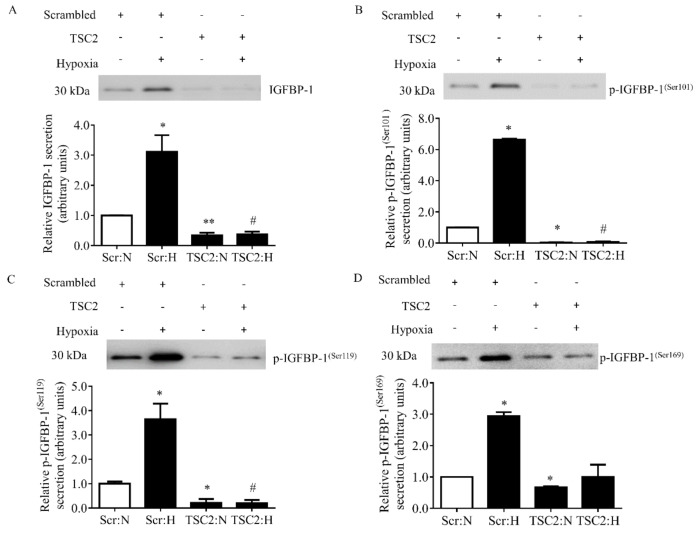
The effect of TSC2 silencing in hypoxia on IGFBP-1 secretion and phosphorylation in decidualized primary HESCs. Decidualized primary HESCs were transfected with TSC2 or Scramble (Scr) siRNA, and then cells were cultured in normoxia (N) (incubator air) or in hypoxia (H) (1% O_2_). Representative Western blots of IGFBP-1 secretion (**A**) and p-IGFBP-1^(Ser101)^ (**B**), p-IGFBP-1^(Ser119)^ (**C**), and p-IGFBP-1^(Ser169)^ (**D**) using equal volume of CM (36 µL). The Western blot data are summarized in the bar graphs (n = 4). Values are given as mean + SEM. * or #, *p* < 0.05; **, *p* = 0.001–0.05 versus control as per one-way ANOVA Tukey’s multiple comparisons test. * = significant difference compared to HESCs transfected with Scr siRNA in normoxia; # = significant difference compared to HESCs transfected with Scr siRNA in hypoxia.

Activation of mTORC1 by TSC2 silencing led to significant reduction in IGFBP-1 secretion (−65%, *p* = 0.001) and phosphorylation at Ser101 (−97%, *p* = 0.024), Ser 119 (−79%, *p* = 0.023) and Ser169 (−32%, *p* = 0.018) in normoxia compared to Scr siRNA (Figure 7A–D). These data demonstrate that mTORC1 signaling is a negative regulator of both IGFBP-1 secretion and phosphorylation.

Hypoxia inhibited mTORC1 (Figure 2) and induced IGFBP-1 secretion and phosphorylation (Figure 3). Using scramble siRNA, we confirmed that IGFBP-1 secretion and phosphorylation was increased in hypoxia (Figure 7). We further assessed the effect of TSC2 silencing on changes in IGFBP-1 secretion and phosphorylation under hypoxia. TSC2 silencing in hypoxia prevented mTORC1 inhibition caused by hypoxia (Figure 6) and subsequently prevented the induction of IGFBP-1 secretion (−88%, *p* = 0.014) and phosphorylation at Ser101 (−99%, *p* = 0.021) and Ser 119 (−95%, *p* = 0.014) due to hypoxia (Figure 7A–D). The gene silencing data with TSC2 siRNA mechanistically confirmed that mTORC1 signaling is involved in mediating the effects of hypoxia on IGFBP-1 secretion and phosphorylation. Overall, these findings provide evidence that mTORC1 signaling plays a critical role in regulating IGFBP-1 secretion and phosphorylation in response to hypoxia.

### 3.11. Targeted PRM-MS Analyses Indicates Reduction of IGFBP-1 Phosphorylation in Decidualized HESCs Treated with TSC2 siRNA

**Figure 8:**
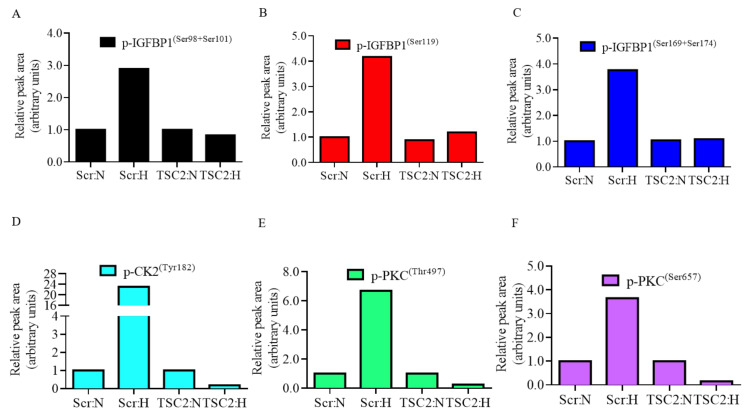
PRM-MS to monitor relative site-specific phosphorylation in response to TSC2 silencing in hypoxia. PRM-MS analysis for detection of relative phosphorylated IGFBP-1 at phosphorylation sites p-IGFBP-1^(Ser98+Ser101)^ (**A**), p-IGFBP-1^(Ser119)^ (**B**), and p-IGFBP-1^(Ser169+Ser174)^ (**C**) in decidualized primary HESCs transfected with TSC2 or Scramble (Scr) siRNA in normoxic (N) and hypoxic (H) conditions. Phosphorylation of IGFBP-1 co-immunoprecipitated CK2 and PKC kinases was also monitored to determine activation status; p-CK2^(Tyr182)^ (**D**), p-PKC^(Thr497)^ (**E**), and p-PKC^(Ser657)^ (**F**) were individually monitored in TSC2 or Scr siRNA conditions. Data was collected using a Q-Exactive hybrid quadrupole-Orbitrap mass spectrometer. Values are displayed as relative peak area of total detected transitions and normalized by an internal IGFBP-1 peptide.

PRM-MS analysis confirmed the effect of TSC2 silencing on IGFBP-1 phosphorylation under hypoxia (Figure 8). The data in Figure 8A–C shows TSC2 silencing in hypoxia relatively inhibited IGFBP-1 phosphorylation for both the singly phosphorylated site, pSer119 (−298%), and dually phosphorylated sites, pSer98+Ser101 (−206%) and pSer169 + Ser174 (−268%).

### 3.12. TSC2 Silencing Prevented the Autophosphorylation of Protein Kinases, CK2 and PKC, in Decidualized HESCs in Hypoxia

Using PRM-MS analyses, we determined that hypoxia treatment led to increased autophosphorylation of CK2 and PKC consistent with increased site-specific phosphorylation in decidualized HESCs. Figure 8D–F shows that hypoxia increased autophosphorylation of p-CK2 (Tyr182, +2206%) and PKC (Thr497, +569%; Ser657, +265%) in HESCs transfected with scramble siRNA. Importantly, TSC2 silencing in hypoxia prevented autophosphorylation of CK2 and PKC, which remained similar to Scr normoxia. These data suggest that mTORC1 signaling regulates the activation of CK2 and PKC in hypoxia by modulating their autophosphorylation levels.

## 4. Discussion

By isolating and characterizing HESCs from first-trimester placenta, we demonstrate, for the first time, that in vitro decidualization of primary HESCs results in markedly increased IGFBP-1 phosphorylation which is further induced by hypoxia, particularly at dually phosphorylated Ser169+Ser174 sites. In addition, selective activation of mTORC1 signaling by TSC2 silencing resulted in decreased IGFBP-1 secretion and site-specific phosphorylation, implicating mTORC1 as a key regulator of the IGFBP-1 system in these cells. TSC2 silencing prevented hypoxia-mediated increase in IGFBP-1 phosphorylation, confirming mechanistically that decidual IGFBP-1 hyperphosphorylation in hypoxia occurs via mTORC1 signaling. The effects of TSC2 gene silencing on IGFBP-1 site-specific phosphorylation were additionally confirmed using targeted PRM-MS analysis. Moreover, PRM-MS data also demonstrated that hypoxia increased p-CK2 (Tyr182), p-PKC (Thr497), and p-PKC (Ser657) autophosphorylation, suggesting increased kinase activation, while TSC2 silencing prevented the effects of hypoxia on kinase activation.

Interestingly, our discovery of doubly phosphorylated peptide Ser169+Ser174, in primary decidualized HESCs treated with hypoxia, matches our findings of hyperphosphorylation of pSer174 in combination with pSer169 in maternal plasma of women with a pregnancy complicated by FGR [25]. These data raise the possibility that the source of dual phosphorylation at Ser169+Ser174 in maternal plasma is the decidua and may have implications in prediction of FGR pregnancies. We propose that decidual mTORC1 links oxygen availability and IGF-1 signaling to placental insufficiency and impaired fetal growth by regulating IGFBP-1 secretion and phosphorylation.

While both IGF-1 and IGF-2 are important in fetoplacental growth, low maternal IGF-1 has been associated with low birth weight in human pregnancies. This is likely to be related to observations that maternal IGF-1 promotes placental development and transfer of nutrients to the fetus [68]. In addition, placental IGF-1R signaling is inhibited in FGR pregnancies [69,70,71]. By modulating interaction of IGF-1 with its receptor IGF-1R, IGFBP-1 plays a key role in controlling bioactivity of its ligand [20]. In general agreement with previous reports [25,31,32,64], in this study we show that IGF-1 bioactivity is greatly attenuated by hypoxia-induced IGFBP-1 hyperphosphorylation in decidualized HESCs. This implies that increase in IGFBP-1 phosphorylation in decidualized HESCs may negatively regulate IGF-1R signaling by inhibiting IGF-1 function.

Using HepG2 cells as a model of human fetal hepatocytes, we reported, for the first time, that site-specific increase in IGFBP-1 phosphorylation inhibits IGF-1 dependent biological activity in response to hypoxia [45]. Considering that transformed cell lines may not represent the biology of primary fetal hepatocytes, we utilized primary baboon hepatocytes and confirmed these findings [31]. In comparison, to assess the status of maternal IGFBP-1, we used human placenta collected at delivery and reported that decidual IGFBP-1 phosphorylation is increased and mTOR is inhibited in human FGR pregnancies [25]. Utilizing a decidualized immortalized cell line (HIESC), we demonstrated that IGFBP-1 phosphorylation is increased in hypoxia and leucine deprivation in vitro [43,52]. We treated the HIESC with pharmacological inhibitors and activators, and our data showed involvement of mTOR and AAR signaling in regulation of decidual IGFBP-1 phosphorylation [43]. These data, however, lacked rigor because two different cell lines (HESCs from ATCC and HIESC) and pharmacological inhibitor rapamycin had been used instead of primary cell and gene targeting approaches. Furthermore, immortalized cell lines may display unique attributes or respond differently compared to primary cells. Immortalized cell lines may have karyotype abnormalities after serial passaging [72], lack physiological responsiveness to progesterone stimulation [73,74,75], or do not express the progesterone receptor (PR), a hallmark protein of primary HESCs that directly stimulates IGFBP-1 transcription [76,77].

Primary HESCs recapitulate some of the key structural, proliferative, and secretory characteristics of decidual cells in vivo [78], and may, therefore, be more physiologically relevant than cell lines to better understand the signaling mechanisms that result in FGR. The mTOR pathway responds to changes in the levels of nutrients, oxygen, and growth factors, such as IGF-1, and, when activated, mTORC1 promotes protein translation, proliferation, and anti-apoptotic activity in most cell types [5,6]. Here, we establish, for the first-time, a mechanistic link between hypoxia, mTORC1 inhibition, and increased IGFBP-1 phosphorylation in in vitro decidualized primary HESCs.

Decidua is subjected to endocrine regulation during pregnancy. For decidualization of stromal cells in vitro, progesterone signaling via the PR is essential and is a prerequisite to successful implantation [50]. Activation of the cAMP second messenger pathway induces diverse transcription factors that are capable of modulating PR, thus sensitizing stromal cells to progesterone action [79]. In the present study, we successfully decidualized the primary cells in vitro, using conventional 8-Br-cAMP + MPA treatments [13,51], that resulted in induced secretion and phosphorylation of IGFBP-1 at three functional sites (Ser 101, 119, and 169). We treated the primary HESCs isolated from early pregnancy placenta with hypoxia. Optimal conditions for hypoxia in cultured decidualized HESCs have, to the best of our knowledge, not been defined. It is believed that 1–3% (term) or 1% oxygen (first-trimester) in vitro reflects in vivo trophoblast hypoxia [80]. We used similar oxygen levels to induce hypoxia in cultured primary decidual HESCs in the current study.

Other studies have reported that placental mTOR signaling is inhibited in pregnancies complicated by FGR due to uteroplacental insufficiency [69,70,81,82]. Hypoxia suppresses mTORC1 signaling. However, a direct link between regulation of decidual IGFBP-1 phosphorylation and the mTORC1 signaling in hypoxia has not been established. It is plausible that tissue levels of nutrients, such as glucose and oxygen (which modulate mTORC1 activity), are decreased in the decidua subjected to placental insufficiency. In the current study, we demonstrated that TSC2 silencing prevented hypoxia-mediated increase in IGFBP-1 phosphorylation in primary HESCs. Furthermore, using PRM-MS we demonstrate for the first time that hypoxia-induced CK2 and PKC autophosphorylation may be regulated via mTORC1 inhibition. Considering that the level of kinase autophosphorylation parallels IGFBP-1 phosphorylation, following TSC2 knock down and hypoxia, these data suggest that CK2 and PKC may be influencing the status of IGFBP-1 phosphorylation. We recently determined that interaction of PKC with protein kinase CK2 and activation of PKC under leucine deprivation mediate fetal hepatic IGFBP-1 hyperphosphorylation. Using HepG2 cells, we determined the nutrient-sensing role of PKC in regulating the phosphorylation of IGFBP-1 [83]. The findings in this study imply that regulation of decidual IGFBP-1 phosphorylation similarly involves activation of PKC.

These findings in primary cells overcome some of the limitations of immortalized cells [84], confirming mechanistically, that decidual IGFBP-1 phosphorylation in hypoxia is mediated by mTORC1 signaling. These data suggest that inhibition of decidual mTORC1 signaling in FGR could potentially result in reduced/compromised IGF-1 action and may contribute to reduced cell growth and proliferation limiting placental development in FGR pregnancies. 

There are nonetheless limitations of this study. It is impossible to model the complexity of the FGR pathophysiology in vitro. Thus, one limitation of our study is that we use a reductionistic approach focusing specifically on selected pathways and it is acknowledged that multiple mechanisms are likely to be important in the development of FGR in vivo. Moreover, although we demonstrated that the activation of CK2 and PKC in hypoxia involves mTORC1 signaling, the mechanisms by which mTORC1 regulates these two kinases remain to be established.

One of the strengths of the study is that we report novel mechanistic information using a physiologically relevant primary HESC- culture to establish the cause-and-effect between mTORC1 activation, regulation of site-specific phosphorylation of IGFBP-1 and protein kinases CK2 and PKC activation. Additionally, the use of PRM-MS analysis is an innovative approach in the current study to identify relative changes in novel and dual phosphorylation at specific IGFBP-1 sites, and for assessment of kinase activation status. Furthermore, in this study we show that IGF-1 bioactivity is greatly attenuated by hypoxia-induced IGFBP-1 hyperphosphorylation in decidualized HESCs. The study provides insight to the biological effects of decidual IGFBP-1 hyperphosphorylation, demonstrating that it results in more prominent inhibition of IGF-1-mediated IGF-1R signaling which may subsequently affect fetal growth.

## 5. Conclusions

In conclusion, we propose a model in which decidual mTORC1 signaling constitutes a critical link between oxygen availability and fetal growth, by influencing IGF-1 function. The proposed work will improve our mechanistic understanding of the molecular link between reduced oxygen availability and restricted fetal growth in placental insufficiency.

## Data Availability

Data is contained within the article.

## Supplementary Materials

The following are available online at https://www.mdpi.com/article/10.3390/biom11091382/s1. IGFBP1_isolationlist.
