# Supplementary material for: Mechanistic Target of Rapamycin Complex 1 Signaling Links Hypoxia to Increased IGFBP-1 Phosphorylation in Primary Human Decidualized Endometrial Stromal Cells
Source: Biomolecules. 2021 Sep 18;11(9):1382. doi: 10.3390/biom11091382 (PMC8471256; doi:10.3390/biom11091382)
Supplement: Supplementary file 1 [file biomolecules-11-01382-s001.zip › biomolecules-1359365-supplementary.pdf]

IGFBP1_isolationlist

Mass [m/z]	CS [z]	Polarity	NCE	Comment
**765.922989**	2	Positive	27	ALPGEQQPLHALTR (light)
**773.319775**	3	Positive	27	AQETS[+80.0]GEEIS[+80.0]KFYLPNCNK (light)
**746.664331**	3	Positive	27	AQETSGEEIS[+80.0]KFYLPNCNK (light)
**746.664331**	3	Positive	27	AQETS[+80.0]GEEISKFYLPNCNK (light)
**1079.50969**	2	Positive	27	AQETSGEEISKFYLPNCNK (light)
**720.008888**	3	Positive	27	AQETSGEEISKFYLPNCNK (light)
**1464.09139**	2	Positive	27	DASAPHAAEAGSPES[+80.0]PES[+80.0]TEITEEELL (light)
**976.396683**	3	Positive	27	DASAPHAAEAGSPES[+80.0]PES[+80.0]TEITEEELL (light)
**1424.10822**	2	Positive	27	DASAPHAAEAGSPESPES[+80.0]TEITEEELL (light)
**949.741239**	3	Positive	27	DASAPHAAEAGSPESPES[+80.0]TEITEEELL (light)
**1424.10822**	2	Positive	27	DASAPHAAEAGSPES[+80.0]PESTEITEEELL (light)
**949.741239**	3	Positive	27	DASAPHAAEAGSPES[+80.0]PESTEITEEELL (light)
**923.085796**	3	Positive	27	DASAPHAAEAGSPESPESTEITEEELL (light)
**430.52041**	3	Positive	27	DNFHLMAPSEE (light)
**765.922989**	2	Positive	27	ALPGEQQPLHALTR (light)
**1079.50969**	2	Positive	27	AQETSGEEISKFYLPNCNK (light)
**430.52041**	3	Positive	27	DNFHLMAPSEE (light)
**685.260143**	2	Positive	27	DNFHLMAPS[+80.0]EE (light)
**457.175854**	3	Positive	27	DNFHLMAPS[+80.0]EE (light)
**433.210189**	2	Positive	27	CARGLSCR (light)
**289.142551**	3	Positive	27	CARGLSCR (light)
**473.193354**	2	Positive	27	CARGLS[+80.0]CR (light)
**315.797995**	3	Positive	27	CARGLS[+80.0]CR (light)
**859.406042**	3	Positive	27	DFIWGFGKQGFQCQVCCFVVHK (light)
**485.216556**	3	Positive	27	RCHEFVTFSCPGA (light)
**960.12063**	3	Positive	27	IHTYGSPTFCDHCGSLLYGLIHQGMK (light)
**937.939141**	2	Positive	27	DHCGSLLYGLIHQGMKC (light)
**663.312202**	3	Positive	27	DMNVHKQCVINVPSLCGM (light)
**937.433888**	2	Positive	27	QCVINVPSLCGMDHTEK (light)
**970.499258**	2	Positive	27	STLNPQWNESFTFKLK (light)
**856.737234**	3	Positive	27	DFMGSLSFGVSELMKMPASGWYK (light)

**962.121458**	3	Positive	27	MPASGWYKLLNQEEGEYYNVPIPEG (light)
**982.46782**	2	Positive	27	LLNQEEGEYYNVPIPEG (light)
**944.972353**	2	Positive	27	PPFLTQLHSCFQTVDR (light)
**852.461415**	3	Positive	27	FKEPQAVFYAAEISIGLFFLHK (light)
**978.489087**	2	Positive	27	GKYSEVFEAINITNNEK (light)
**652.661817**	3	Positive	27	GKYSEVFEAINITNNEK (light)
**885.930873**	2	Positive	27	YSEVFEAINITNNEK (light)
**590.956341**	3	Positive	27	YSEVFEAINITNNEK (light)
**1098.58098**	2	Positive	27	YSEVFEAINITNNEKVVVK (light)
**732.723076**	3	Positive	27	YSEVFEAINITNNEKVVVK (light)
**564.34278**	2	Positive	27	EIKILENLR (light)
**376.564279**	3	Positive	27	EIKILENLR (light)
**797.469772**	2	Positive	27	ILENLRGGPNIITLA (light)
**531.982273**	3	Positive	27	ILENLRGGPNIITLA (light)
**428.25036**	2	Positive	27	GGPNIITLA (light)
**285.835999**	3	Positive	27	GGPNIITLA (light)
**655.887552**	2	Positive	27	GGPNIITLADIVK (light)
**437.594127**	3	Positive	27	GGPNIITLADIVK (light)
**514.790381**	2	Positive	27	DIVKDPVSR (light)
**343.529346**	3	Positive	27	DIVKDPVSR (light)
**948.484139**	2	Positive	27	DPVSRTPALVFEHVNNT (light)
**632.658518**	3	Positive	27	DPVSRTPALVFEHVNNT (light)
**671.343509**	2	Positive	27	TPALVFEHVNNT (light)
**447.898098**	3	Positive	27	TPALVFEHVNNT (light)
**866.438669**	2	Positive	27	TPALVFEHVNNTDFK (light)
**577.961538**	3	Positive	27	TPALVFEHVNNTDFK (light)
**628.829703**	2	Positive	27	DFKQLYQTLT (light)
**419.55556**	3	Positive	27	DFKQLYQTLT (light)
**572.779679**	2	Positive	27	QLYQTLTDY (light)
**382.188878**	3	Positive	27	QLYQTLTDY (light)
**745.889237**	2	Positive	27	DIRFYMYEILK (light)
**497.59525**	3	Positive	27	DIRFYMYEILK (light)
**553.783178**	2	Positive	27	FYMYEILK (light)
**369.524544**	3	Positive	27	FYMYEILK (light)

**645.843767**	2	Positive	27	FYMYEILKAL (light)
**430.89827**	3	Positive	27	FYMYEILKAL (light)
**526.781501**	2	Positive	27	DVKPHNVMI (light)
**351.523426**	3	Positive	27	DVKPHNVMI (light)
**692.830577**	2	Positive	27	PHNVMIDHEHR (light)
**462.22281**	3	Positive	27	PHNVMIDHEHR (light)
**1154.06329**	2	Positive	27	LIDWGLAEFYHPGQEYNVR (light)
**769.711282**	3	Positive	27	LIDWGLAEFYHPGQEYNVR (light)
**1040.97922**	2	Positive	27	DWGLAEFYHPGQEYNVR (light)
**694.321906**	3	Positive	27	DWGLAEFYHPGQEYNVR (light)
**1247.59855**	2	Positive	27	DWGLAEFYHPGQEYNVRVASR (light)
**832.068128**	3	Positive	27	DWGLAEFYHPGQEYNVRVASR (light)
**533.302592**	2	Positive	27	YFKGPELLV (light)
**355.87082**	3	Positive	27	YFKGPELLV (light)
**664.315571**	2	Positive	27	GPELLVDYQMY (light)
**443.212806**	3	Positive	27	GPELLVDYQMY (light)
**599.242072**	2	Positive	27	DYQMYDYSL (light)
**399.830473**	3	Positive	27	DYQMYDYSL (light)
**499.745776**	2	Positive	27	KEPFFHGH (light)
**333.499609**	3	Positive	27	KEPFFHGH (light)
**631.764894**	2	Positive	27	EPFFHGHDNY (light)
**421.512355**	3	Positive	27	EPFFHGHDNY (light)
**511.748713**	2	Positive	27	DNYDQLVR (light)
**341.501567**	3	Positive	27	DNYDQLVR (light)
**471.790184**	2	Positive	27	DQLVRIAK (light)
**314.862548**	3	Positive	27	DQLVRIAK (light)
**415.752735**	2	Positive	27	IAKVLGTE (light)
**277.504249**	3	Positive	27	IAKVLGTE (light)
**455.231833**	2	Positive	27	VLGTEDLY (light)
**303.823647**	3	Positive	27	VLGTEDLY (light)
**510.261456**	2	Positive	27	YNIELDPR (light)
**340.510063**	3	Positive	27	YNIELDPR (light)
**477.267407**	2	Positive	27	DILGRHSR (light)
**318.51403**	3	Positive	27	DILGRHSR (light)

**1089.53998**	2	Positive	27	WERFVHSENQHLVSPEAL (light)
**726.695743**	3	Positive	27	WERFVHSENQHLVSPEAL (light)
**853.928468**	2	Positive	27	FVHSENQHLVSPEAL (light)
**569.621404**	3	Positive	27	FVHSENQHLVSPEAL (light)
**1041.51818**	2	Positive	27	FVHSENQHLVSPEALDFL (light)
**694.681211**	3	Positive	27	FVHSENQHLVSPEALDFL (light)
**542.286328**	2	Positive	27	DHQSRLTAR (light)
**361.859977**	3	Positive	27	DHQSRLTAR (light)
**1028.02204**	2	Positive	27	LTAREAMEHPYFYTVVK (light)
**685.683787**	3	Positive	27	LTAREAMEHPYFYTVVK (light)
**807.387059**	2	Positive	27	EAMEHPYFYTVVK (light)
**538.593798**	3	Positive	27	EAMEHPYFYTVVK (light)
**1042.49893**	2	Positive	27	EAMEHPYFYTVVKDQAR (light)
**695.335046**	3	Positive	27	EAMEHPYFYTVVKDQAR (light)
**1194.04645**	2	Positive	27	LIDWGLAEFY[+80.0]HPGQEYNVR (light)
**796.366725**	3	Positive	27	LIDWGLAEFY[+80.0]HPGQEYNVR (light)
**1080.96239**	2	Positive	27	DWGLAEFY[+80.0]HPGQEYNVR (light)
**720.977349**	3	Positive	27	DWGLAEFY[+80.0]HPGQEYNVR (light)
**1287.58172**	2	Positive	27	DWGLAEFY[+80.0]HPGQEYNVRVASR (light)
**858.723572**	3	Positive	27	DWGLAEFY[+80.0]HPGQEYNVRVASR (light)
**1247.59855**	2	Positive	27	DWGLAEFYHPGQEYNVRVASR (light)
**832.068128**	3	Positive	27	DWGLAEFYHPGQEYNVRVASR (light)
**1287.58172**	2	Positive	27	DWGLAEFYHPGQEYNVRVAS[+80.0]R (light)
**858.723572**	3	Positive	27	DWGLAEFYHPGQEYNVRVAS[+80.0]R (light)
**627.792794**	2	Positive	27	DGVTTRTFCGTP (light)
**418.864288**	3	Positive	27	DGVTTRTFCGTP (light)
**1174.06939**	2	Positive	27	TFCGTPDYIAPEIIAYQPYGK (light)
**783.048683**	3	Positive	27	TFCGTPDYIAPEIIAYQPYGK (light)
**1362.65065**	2	Positive	27	DQSDFEGFSYVNPQFVHPILQSAV (light)
**908.769525**	3	Positive	27	DQSDFEGFSYVNPQFVHPILQSAV (light)
**1197.59188**	2	Positive	27	DFEGFSYVNPQFVHPILQSAV (light)
**798.730342**	3	Positive	27	DFEGFSYVNPQFVHPILQSAV (light)
**667.77596**	2	Positive	27	DGVTTRT[+80.0]FCGTP (light)
**445.519732**	3	Positive	27	DGVTTRT[+80.0]FCGTP (light)

**1214.05255**	2	Positive	27	T[+80.0]FCGTPDYIAPEIIAYQPYGK (light)
**809.704127**	3	Positive	27	T[+80.0]FCGTPDYIAPEIIAYQPYGK (light)
**1402.63382**	2	Positive	27	DQSDFEGFS[+80.0]YVNPQFVHPILQSAV (light)
**935.424969**	3	Positive	27	DQSDFEGFS[+80.0]YVNPQFVHPILQSAV (light)
**1237.57504**	2	Positive	27	DFEGFS[+80.0]YVNPQFVHPILQSAV (light)
**825.385786**	3	Positive	27	DFEGFS[+80.0]YVNPQFVHPILQSAV (light)
